# Site suitability-based spatial-weighted multicriteria analysis for nuclear power plants in Indonesia

**DOI:** 10.1016/j.heliyon.2022.e09088

**Published:** 2022-03-10

**Authors:** Heni Susiati, Moh. Dede, Millary Agung Widiawaty, Arif Ismail, Pande Made Udiyani

**Affiliations:** a Research Center for Assessment of Nuclear Energy System (PKSEN), National Nuclear Energy Agency of Indonesia (BATAN), Jakarta, 12710, Indonesia; b Center for Environment and Sustainability Science, Universitas Padjadjaran, Bandung, West Java, 40132, Indonesia; c Department of Geography Education, Faculty of Social Sciences Education (FPIPS), Universitas Pendidikan Indonesia, Bandung City, West Java, 40154, Indonesia; d Research Center for Nuclear Reactor Technology and Safety (PTKRN), National Nuclear Energy Agency (BATAN), Jakarta, 12710, Indonesia

**Keywords:** AHP, GIS, Nuclear power plants, Suitable locations, West Kalimantan

## Abstract

Nuclear energy is a choice to meet the electricity needs of West Kalimantan, Indonesia to support new industrial areas. Nuclear Power Plants (NPPs) require an appropriate location in terms of biogeophysical, socio-economic, and disaster considerations. This study aims to determine potential sites for NPPs using spatial-weighted multicriteria analysis. GIS and analytical hierarchical processes (AHP) were combined to produce suitable NPPs site. This study showed that West Kalimantan has a very high suitability area covering 16,321.66 Km^2^ (25.81 %) for NPPs. The NPPs are proposed to operate with water reactor coolants, thereby prioritizing areas of about five kilometres from the shoreline. We discovered twelve sites, but only two suitable priority sites for NPPs were found in Ketapang after overlapping with national/regional planning and the existing state. These sites will not significantly impact the environment, socio-economic, and policies, also will support electricity for the new capital region for Indonesia.

## 1 Introduction

Development in West Kalimantan addresses fundamental challenges related to the availability of electricity [1], and a Nuclear Power Plant (NPP) has been the option of renewable energy needs in the regional development plan since 2010 [2]. Indonesia has electrical power supplies derived from coal (50 %), natural gas (29 %), renewable energy (14 %) and petroleum (7 %) plants [3]. Meanwhile, the use of clean energy is necessary to achieve sustainable development goals. The central government supports the development of nuclear power plants through Presidential Regulation Number 5 of 2006, which stipulates that these energy sources can supply 5 % of the nation's electricity needs. This mandate is supported by Law Number 17 of 2007 concerning Long-Term Development Plans from 2005 to 2025 [4]. However, the development of nuclear power plants in Indonesia is still hindered by public acceptance and location suitability. The citizens are unaware of the advantages of these plants over other sources of electrical energy and consider them unsafe, explosive, hazardous waste, with health-threatening radiation [5, 6]. Especially for West Kalimantan, local communities have received socialization on the construction of nuclear power plants and they are very receptive [7, 8].

Electricity development in Indonesia is directed at providing electricity based on non-fossil energy as a long-term goal of national energy diversification and emission control, especially through the use of nuclear and renewable energy [9]. Nuclear power plant sites require the proper formulation of structures, systems, and components to ensure their operations remain safe during normal or abnormal conditions caused by various internal and external threats [10, 11, 12]. According to the International Atomic Energy Agency (IAEA), potential locations can be obtained in stages, starting at the regional level, then filtering via additional characteristics and more detailed criteria [13, 14]. The selection of these plants in Indonesia meets the safety requirements set by the Nuclear Energy Supervisory Agency (BAPETEN). Generally, nuclear power plants should be technically free of and avoid the threat of catastrophic biophysical and human activities, for which engineering technology has been unable to improve management capacity. In addition, these power plants should be constructed at locations that can supply backup electricity and simultaneously channel the appropriate technical restrictions required [15].

The various requirements set by these institutions indicate that selecting a nuclear power plant site must employ a common multicriteria analysis for various development planning or evaluation activities [16]. This analysis adopts decision-making processes by considering each factor's degree of importance in the study location [17]. In an Iranian study, the multicriteria analysis provided greater flexibility and more reliable results for developing future nuclear power plant projects by considering determinants and constraints [18]. The siting of nuclear power plants in Sweden uses various determinants, especially for the safety of reactor operations, such as being distant from settlements, avoiding locations prone to multi-disasters and easy accessibility [19]. Following the unprecedented earthquake and tsunami events in Fukushima, Japan, in 2011, the multicriteria analysis will also facilitate the evaluation of site suitability for nuclear power plants, including land-use factors, earthquakes, population density, and evacuation capacity [20]. In multicriteria analysis, the formulation of these factors must include the provision of weights through weighted linear combination (WLC), analytical hierarchy process (AHP), or expert judgment [21, 22, 23]. Meanwhile, the weighed factors will be easy to analyze using a spatial approach through the geographic information system (GIS) [24].

Previous studies on site suitability for nuclear power plants have been conducted in many Indonesian regions, such as Gunung Muria (Jepara Regency, Central Java), Kramatwatu (Banten), Bangka Island, West Nusa Tenggara, and East Kalimantan [25, 26, 27, 28, 29]. Specifically for nuclear power plants at Gunung Muria and Bangka Island, both have started to the feasibility study and site vicinity [30]. However, they weighed each parameter only based on expert judgment or previous re-search syntheses. This research is different from another assessment of potential sites for nuclear power plants in Indonesia which only uses scores and weights. This research emphasizes the integration of AHP and GIS which is able to accommodate the characteristics and complexities in West Kalimantan. Therefore, this study aims to determine potential nuclear power plant sites using spatial-weighted multicriteria analysis in West Kalimantan, Indonesia. GIS and analytical hierarchy processes (AHP) were combined to generate the site suitability by considering the biophysical, socio-economic, disaster, and constraint dimensions, according to the geographical characteristics of West Kalimantan. Consequently, this study can be input for stakeholders to realize the first nuclear power plant in Indonesia. West Kalimantan requires a potential site assessment for an adequate nuclear power plant considering that in the 2020–2030 period, the region must immediately begin a detailed environmental assessment.

## 2 Materials and methods

### 2.1 Study location and data acquisition

**Figure 1:**
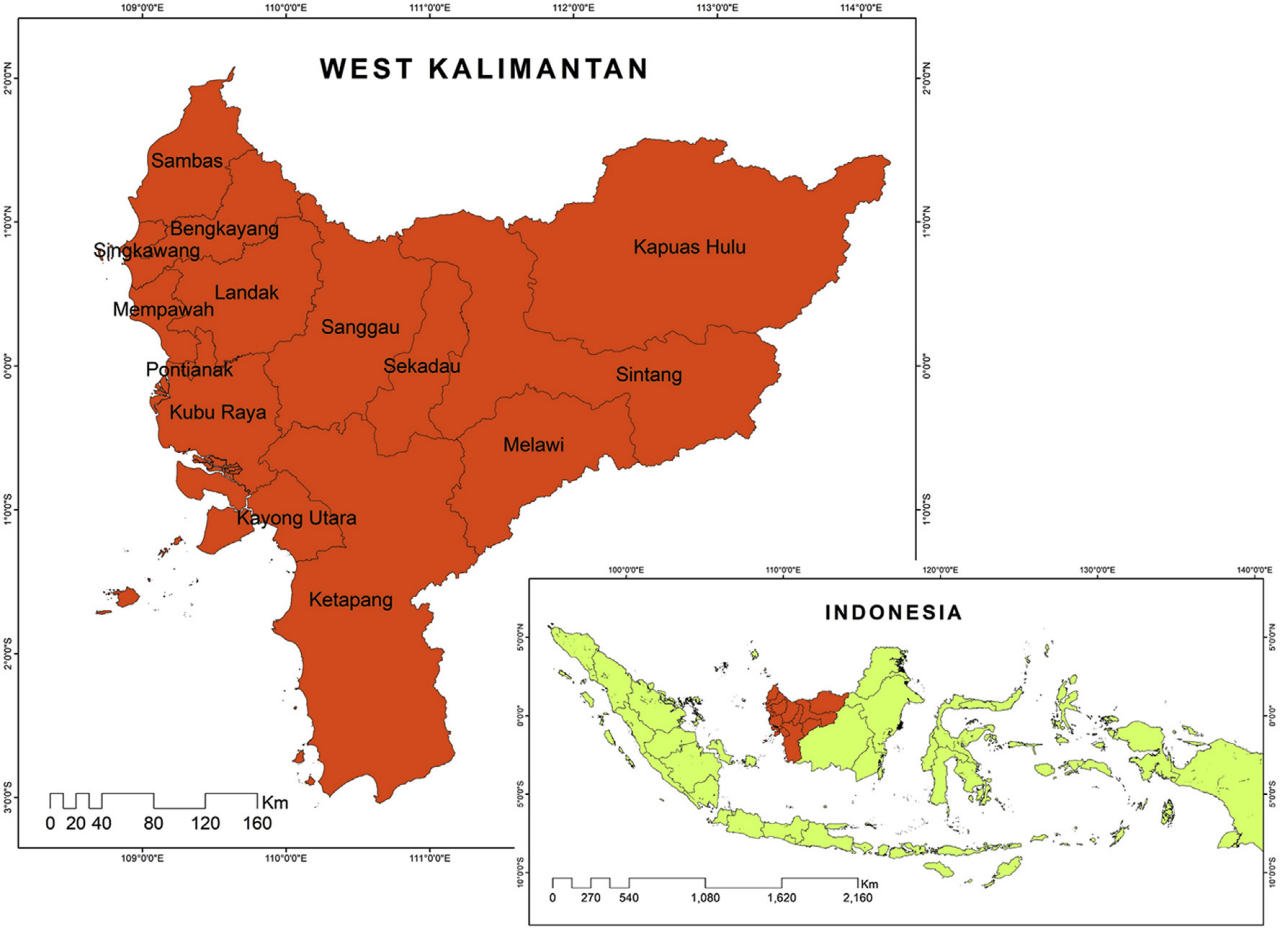
Research location in West Kalimantan.

This study occurred in the West Kalimantan province, Indonesia, which is bordered by Sarawak (Malaysia), South Kalimantan, Karimata Strait, and Java Sea (Figure 1). West Kalimantan is divided into two cities and six districts, Sambas, Bengkayang, Singkawang, Pontianak, Mempawah, Kubu Raya, Kayong Utara, Ketapang, and has a 5.41 million population [31]. This province has become an area projected for building re-newable electrical energy sources to support various development projects, such as nuclear and solar power plants [1, 32, 33]. Subsequently, this study employed 14 parameters to determine the appropriate sites for nuclear power plants based on criteria from IAEA, BAPETEN, and BATAN, as shown in Table 1. These parameters consist of 9 biogeophysical, 4 socio-economic, and 1 disaster aspects, which should be presented in geospatial data for easy integration.

**Table 1:** Data sources for generating spatial parameters of nuclear power plants.

Aspect	Parameter	Data Sources	Method
Biogeophysical	Physiographic	Copernicus DEM (European Space Agency)	Reclassification
Biogeophysical	Land slope	Copernicus DEM (European Space Agency)	Slope analysis
Biogeophysical	Groundwater	Indonesian Ministry of Energy and Mineral Resources (KESDM RI)	Not applicable
Biogeophysical	Soils	UN Food and Agricultural Organization (FAO)	Not applicable
Biogeophysical	Rainfall	CHIRPS (University of California at Santa Barbara)	Downscaling; reclassification
Biogeophysical	Climate	Indonesian Agency for Meteorology, Climatology, and Geophysics (BMKG)	Not applicable
Biogeophysical	Land use and land cover	Indonesian Ministry of Environment and Forestry (KLHK RI)	Not applicable
Biogeophysical	Landsystem	Regional Physical Planning Project for Transmigration of Indonesia (RePPProT)	Not applicable
Socio-economic	Distance from settlement	Indonesian Ministry of Environment and Forestry (KLHK RI)	Buffering
Socio-economic	Accessibility	Indonesian Ministry of Public Works and Housing (KemenPUPR RI)	Buffering
Socio-economic	Central Business District	Indonesian Geospatial Information Agency (BIG)	Buffering
Socio-economic	Vital and dangerous infrastructures	Indonesian Ministry of Industry (Kemenperin RI); Indonesian Ministry of Transportation (Kemenhub RI); Indonesian Ministry of Energy and Mineral Resources (KESDM RI)	Buffering
Biogeophysical	Geological structures	Indonesian Ministry of Energy and Mineral Resources (KESDM RI)	Buffering
Disaster	Disaster risk	Indonesian Ministry of Environment and Forestry (KLHK RI)	Reclassification

A score was accorded to each parameter to aid data analysis and information presentation. As shown in Table 2, each parameter is divided into five scores readjusted according to characteristics and geographical data variations, based on the regulations and results of previous studies. Obtaining required information from some data necessitates treatment, especially buffering analysis. Restricted areas currently designated as protected, cultivation rights, production forests, and other regions not allocated for any human-physical development were considered through regional and national regulations in determining the nuclear power plant site. Also considered was the possibility of locating these plants near the coastline to facilitate the use of more environmentally friendly reactor coolants, such as water, gas, or sodium [43, 44, 45, 46].

**Table 2:** Score for the suitability parameters of nuclear power plants.

Parameter	Score	Information	Source
Physiographic	5 (0–70 m); 4 (70–210 m); 3 (210–350 m); 2 (350–550 m); 1 (>550 m)	Physiographic is only refer to the concept of elevation above sea level	[34]
Land slope	5 (0–2 %); 4 (3–7 %); 3 (8–15 %); 2 (16–25 %); 1 (>26 %)	Land slope is ratio of elevation and distance between different places	[35]
Groundwater	5 (high); 4 (medium); 3 (locally); 2 (low); 1 (scarce area)	Groundwater is water beneath Earth's surface between rock and soil layer	[36]
Soils	5 (fluvisols, regosols); 4 (acrisols, arenosols, cambisols, podzols); 3 (gleysols); 2 (ferralsols); 1 (histosols, nitosols)	Soil is the Earth's surface layer that is composed of mineral and organic materials	[37]
Rainfall	5 (>2000 mm/yr); 4 (1500–2000 mm/yr); 3 (1000–1500 mm/yr); 2 (500–1000 mm/yr); 1 (0–500 mm/yr)	Rainfall is amount of water that falls on the surface during a certain period measured in millimetre above the horizontal surface	[38]
Climate	5 (A); 4 (B); 3 (C); 2 (D); 1 (E)	Climate is atmosphere condition including temperature, rainfall, wind in wide areas and in long period (20–30 years)	[39]
Land use and land cover	5 (open land); 4 (marsh, scrub); 3 (mining, plantation, industrial forest); 2 (rice field, agriculture, fishpond); 1 (built-up area, forest, water bodies)	Land use and land cover is land utilization by human for certain uses	[28]
Landsystem	5 (coastal flats/plains); 4 (river valley); 3 (montane wetland/other foothills/wetlands); 2 (volcanic ridge/hills/other ridge); 1 (acid/lakes)	Landsystem is information compiled based on geological factors and the formation process with climatic influences to produce land unit	[40]
Distance from settlement	5 (>20 Km); 4 (15–20 Km); 3 (10–15 Km); 2 (5–10 Km); 1 (0–5 Km)	Radius from nearest settlement	[41]
Accessibility	5 (>20 Km); 4 (15–20 Km); 3 (10–15 Km); 2 (5–10 Km); 1 (0–5 Km)	Distance from main roads (artery and collector)	[41]
Central business district	5 (>20 Km); 4 (15–20 Km); 3 (10–15 Km); 2 (5–10 Km); 1 (0–5 Km)	Distance from the economic centre at the district/city level	[41]
Vital and dangerous infrastructures	5 (>20 Km); 4 (15–20 Km); 3 (10–15 Km); 2 (5–10 Km); 1 (0–5 Km)	Consists of a refueling station, oil/gas refinery, main harbour and airport	[41]
Geological structures	5 (>20 Km); 4 (15–20 Km); 3 (10–15 Km); 2 (5–10 Km); 1 (0–5 Km)	Distance from faults in the Earth's surface	[28]
Disaster risk	5 (0.20–0.30); 4 (0.31–0.40); 3 (0.41–0.50); 2 (0.51–0.60); 1 (0.61–0.70)	The risk of multiple disasters caused by natural and human factors	[42]

### 2.2 Site suitability-based spatial-weighted multicriteria analysis

**Figure 2:**
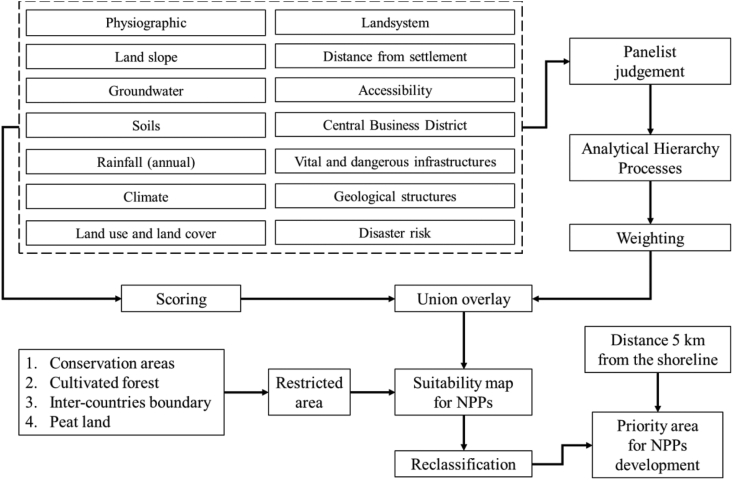
Framework of site suitability for nuclear power plants.

**Figure 3:**
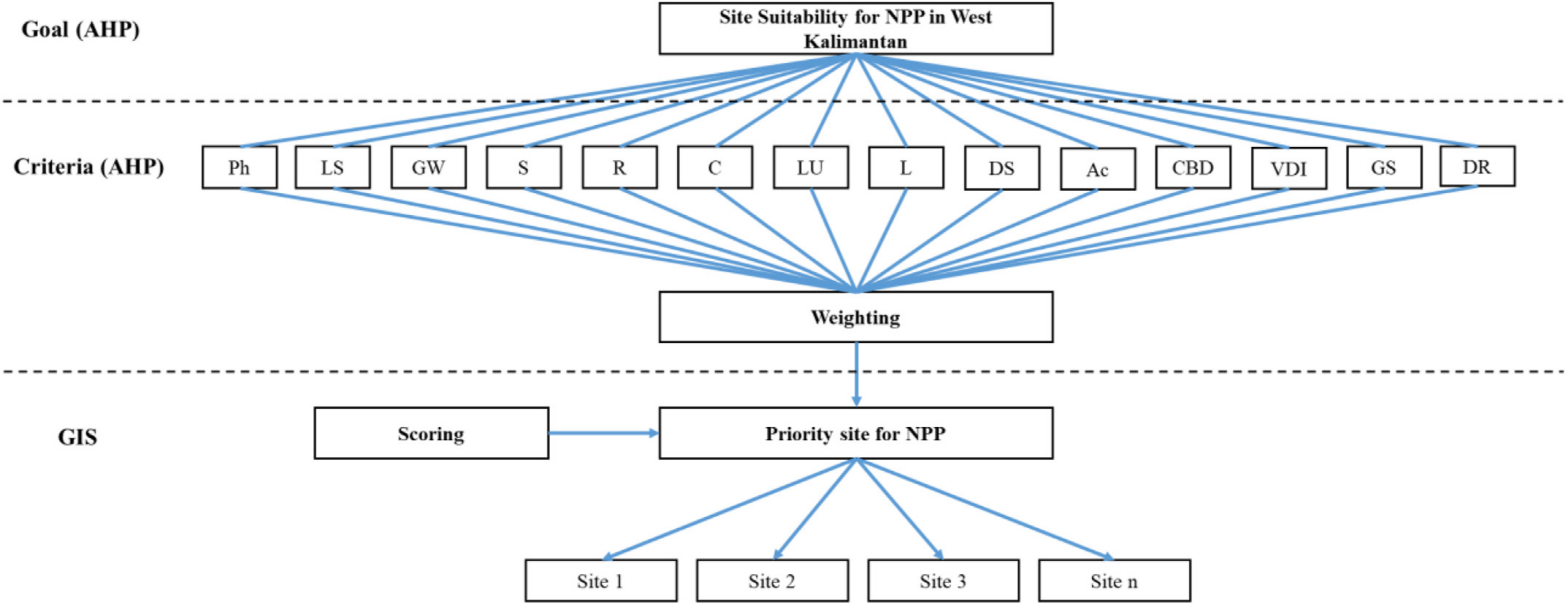
Hierarchical arrangement of suitability for nuclear power plants (Ph = Physiographic; LS = Land slope; GW = Groundwater; S= Soils; R = Rainfall; C= Climate; LU = Land use and land cover; L = Landsystem; DS = Distance from settlement; Ac= Accessibility; CBD = Central Business District; VDI = Vital and dangerous infrastructures; GS = Geological structures; DR = Disaster risk).

The framework analysis of this study is illustrated in Figure 2. Parameters for determining the suitability of nuclear power plants require scores and weights. The scores refer to previous studies, while the weights are derived from the results of the AHP analysis. Overlay analysis facilitates the summation of scores and weights of each parameter. By considering the restricted area and distance from the coast, we can determine the priority locations for nuclear power plants in West Kalimantan. The determination of weights for parameters is crucial for spatial-multicriteria analysis. In this study, the stages comprised 1) identification; 2) hierarchical arrangement (Figure 3); 3) making comparison matrices; 4) performing pairwise comparisons (normalization); 5) weight comparisons; 6) consistency checks [47, 48]. Decision-makers should focus on a hierarchical structure, validity, and inconsistency in evaluating the 14 nuclear power plant site parameters. Valid and consistent numerical weight values can be employed for spatial analysis through GIS using the overlay technique [24]. Meanwhile, the identification and hierarchical arrangement refer to results of discussions by four panelists as shown in Table 3. Panelists have competent backgrounds in geospatial analysis, environmental analysis, decision making, and energy policy. Currently, panelists work in different institutions, as academics from Universitas Pendidikan Indonesia and Universitas Padjadjaran, as well as senior researcher at BATAN.

**Table 3:** Priority for each parameter.

Parameter	Panelist				Average (rounded)	Priority
	I	II	III	IV		
Physiographic	7	6	7	9	7	3
Land slope	7	7	7	9	8	2
Groundwater	4	3	5	9	5	5
Soils	3	5	3	9	5	5
Rainfall	1	1	3	7	3	6
Climate	5	2	3	7	4	3
Land use and land cover	6	8	6	5	6	4
Landsystem	9	6	5	9	7	3
Distance from settlement	9	9	9	9	9	1
Accessibility	7	8	7	5	7	3
Central Business District	9	8	9	4	8	2
Vital and dangerous infrastructures	7	9	9	5	8	2
Geological structures	9	7	7	9	8	2
Disaster risk	8	8	8	9	8	2

Based on the priority value, the analysis stage to obtain the weighting value proceeds to the pairwise comparison or normalization stage (Table 4). Each factor's priority value must be divided by the sum of its columns. Then, an average column should be created horizontally on the right side for the normalized values, which will subsequently be used as a reference for weight comparison [49], this stage is shown in Table 5. The consistency between weights was performed by calculating the consistency index and ratio, and the weights were declared valid and appropriate for analysis of site suitability of nuclear power plants. As shown in Table 6, the weights were multiplied by a constant of 100 and rounded to make calculations easier. The AHP analysis produced an average consistency vector value of 17.00 an index and ratio of 0.00 each, which were declared valid as they were <0.1 [50,51]. After the scores and weights were met, all data were overlaid into one single value, and the overlapped areas were eliminated to avoid occupying the restricted areas mentioned earlier. The level of site suitability referred to the quantile classification because this method can consider the equal number of values as a basis for grouping data [52, 53].

**Table 4:** The pairwise comparison.

Criteria	R	C	GW	S	LU	Ph	L	Ac	LS	DR	CBD	VDI	GS	DS
R	1	3/4	3/5	3/5	1/2	3/7	3/7	3/7	3/8	3/8	3/8	3/8	3/8	1/3
C	1 1/3	1	4/5	4/5	2/3	4/7	4/7	4/7	1/2	1/2	1/2	1/2	1/2	4/9
GW	1 2/3	1 1/4	1	1	5/6	5/7	5/7	5/7	5/8	5/8	5/8	5/8	5/8	5/9
S	1 2/3	1 1/4	1	1	5/6	5/7	5/7	5/7	5/8	5/8	5/8	5/8	5/8	5/9
LU	2	1 1/2	1 1/5	1 1/5	1	6/7	6/7	6/7	3/4	3/4	3/4	3/4	3/4	2/3
Ph	2 1/3	1 3/4	1 2/5	1 2/5	1 1/6	1	1	1	7/8	7/8	7/8	7/8	7/8	7/9
L	2 1/3	1 3/4	1 2/5	1 2/5	1 1/6	1	1	1	7/8	7/8	7/8	7/8	7/8	7/9
Ac	2 1/3	1 3/4	1 2/5	1 2/5	1 1/6	1	1	1	7/8	7/8	7/8	7/8	7/8	7/9
LS	2 2/3	2	1 3/5	1 3/5	1 1/3	1 1/7	1 1/7	1 1/7	1	1	1	1	1	8/9
DR	2 2/3	2	1 3/5	1 3/5	1 1/3	1 1/7	1 1/7	1 1/7	1	1	1	1	1	8/9
CBD	2 2/3	2	1 3/5	1 3/5	1 1/3	1 1/7	1 1/7	1 1/7	1	1	1	1	1	8/9
VDI	2 2/3	2	1 3/5	1 3/5	1 1/3	1 1/7	1 1/7	1 1/7	1	1	1	1	1	8/9
GS	2 2/3	2	1 3/5	1 3/5	1 1/3	1 1/7	1 1/7	1 1/7	1	1	1	1	1	8/9
DS	3	2 1/4	1 4/5	1 4/5	1 1/2	1 2/7	1 2/7	1 2/7	1 1/8	1 1/8	1 1/8	1 1/8	1 1/8	1

Note: Ph = Physiographic; LS = Land slope; GW = Groundwater; S= Soils; R = Rainfall; C= Climate; LU = Land use and land cover; L = Landsystem; DS = Distance from settlement; Ac= Accessibility; CBD = Central Business District; VDI = Vital and dangerous infrastructures; GS = Geological structures; DR = Disaster risk).

**Table 5:** Normalization.

Criteria	R	C	GW	S	LU	Ph	L	Ac	LS	DR	CBD	VDI	GS	DS
R	0.03	0.03	0.03	0.03	0.03	0.03	0.03	0.03	0.03	0.03	0.03	0.03	0.03	0.03
C	0.04	0.04	0.04	0.04	0.04	0.04	0.04	0.04	0.04	0.04	0.04	0.04	0.04	0.04
GW	0.05	0.05	0.05	0.05	0.05	0.05	0.05	0.05	0.05	0.05	0.05	0.05	0.05	0.05
S	0.05	0.05	0.05	0.05	0.05	0.05	0.05	0.05	0.05	0.05	0.05	0.05	0.05	0.05
LU	0.06	0.06	0.06	0.06	0.06	0.06	0.06	0.06	0.06	0.06	0.06	0.06	0.06	0.06
Ph	0.08	0.08	0.08	0.08	0.08	0.08	0.08	0.08	0.08	0.08	0.08	0.08	0.08	0.08
L	0.08	0.08	0.08	0.08	0.08	0.08	0.08	0.08	0.08	0.08	0.08	0.08	0.08	0.08
Ac	0.08	0.08	0.08	0.08	0.08	0.08	0.08	0.08	0.08	0.08	0.08	0.08	0.08	0.08
LS	0.09	0.09	0.09	0.09	0.09	0.09	0.09	0.09	0.09	0.09	0.09	0.09	0.09	0.09
DR	0.09	0.09	0.09	0.09	0.09	0.09	0.09	0.09	0.09	0.09	0.09	0.09	0.09	0.09
CBD	0.09	0.09	0.09	0.09	0.09	0.09	0.09	0.09	0.09	0.09	0.09	0.09	0.09	0.09
VDI	0.09	0.09	0.09	0.09	0.09	0.09	0.09	0.09	0.09	0.09	0.09	0.09	0.09	0.09
GS	0.09	0.09	0.09	0.09	0.09	0.09	0.09	0.09	0.09	0.09	0.09	0.09	0.09	0.09
DS	0.10	0.10	0.10	0.10	0.10	0.10	0.10	0.10	0.10	0.10	0.10	0.10	0.10	0.10

**Table 6:** Weighted sum vector.

Criteria	CH	I	Ak	JT	PL	M	Ls	Ak	L	RB	SR	IV	SG	PD	Sum
CH	0.03	0.03	0.03	0.03	0.03	0.03	0.03	0.03	0.03	0.03	0.03	0.03	0.03	0.03	0.45
I	0.04	0.04	0.04	0.04	0.04	0.04	0.04	0.04	0.04	0.04	0.04	0.04	0.04	0.04	0.60
Ak	0.05	0.05	0.05	0.05	0.05	0.05	0.05	0.05	0.05	0.05	0.05	0.05	0.05	0.05	0.75
JT	0.05	0.05	0.05	0.05	0.05	0.05	0.05	0.05	0.05	0.05	0.05	0.05	0.05	0.05	0.75
PL	0.06	0.06	0.06	0.06	0.06	0.06	0.06	0.06	0.06	0.06	0.06	0.06	0.06	0.06	0.90
M	0.08	0.08	0.08	0.08	0.08	0.08	0.08	0.08	0.08	0.08	0.08	0.08	0.08	0.08	1.05
Ls	0.08	0.08	0.08	0.08	0.08	0.08	0.08	0.08	0.08	0.08	0.08	0.08	0.08	0.08	1.05
Ak	0.08	0.08	0.08	0.08	0.08	0.08	0.08	0.08	0.08	0.08	0.08	0.08	0.08	0.08	1.05
L	0.09	0.09	0.09	0.09	0.09	0.09	0.09	0.09	0.09	0.09	0.09	0.09	0.09	0.09	1.20
RB	0.09	0.09	0.09	0.09	0.09	0.09	0.09	0.09	0.09	0.09	0.09	0.09	0.09	0.09	1.20
SR	0.09	0.09	0.09	0.09	0.09	0.09	0.09	0.09	0.09	0.09	0.09	0.09	0.09	0.09	1.20
IV	0.09	0.09	0.09	0.09	0.09	0.09	0.09	0.09	0.09	0.09	0.09	0.09	0.09	0.09	1.20
SG	0.09	0.09	0.09	0.09	0.09	0.09	0.09	0.09	0.09	0.09	0.09	0.09	0.09	0.09	1.20
PD	0.10	0.10	0.10	0.10	0.10	0.10	0.10	0.10	0.10	0.10	0.10	0.10	0.10	0.10	1.35

To reveal the weight stability of AHP (Table 7), the weight result was tested for sensitivity by comparing it with the Best Worst Method (BWM) as an alternative weighting. Sensitivity aims to know the difference in weight between the alternatives and which parameters are easy to change [54, 55, 56]. BWM is part of the Multicriteria Decision Making (MCDM) methods, it is useful for calculating the weight (importance) of each criterion (parameter) [57, 58]. BWM is a vector-based method, it requires fewer comparisons compared to AHP [59]. A similarity between BWW and AHP is the usage of consistency ratio for weighting results validation [60]. AHP refers to the inconsistency of decision-makers in pairwise comparisons, and BWM is useful to reduce this inconsistency [61].

**Table 7:** The weight of AHP for nuclear power plants suitability.

Parameter	Weight	Parameter	Weight
Physiographic	8	Landsystem	8
Land slope	9	Distance from settlement	10
Groundwater	5	Accessibility	8
Soils	5	Central Business District	9
Rainfall	3	Vital and dangerous infrastructures	9
Climate	4	Geological structures	9
Land use and land cover	6	Disaster risk	9

## 3 Results

West Kalimantan has dominant lowland physiography with many large rivers traversing the area. Muller, Schwaner, Kapuas Atas, and Kelingkang Mountains, which are less than 10 % wide and have a peak of 2278 m above sea level (Mount Baturaya), can be found in the areas bordering Sarawak, as well as Central, North, and East Kalimantan [62]. The flat, sloping area is located on the west close to the sea (coastal zone) and can also be found in the central part of West Kalimantan, known as Kapuas Valley. Physiography and slope play important roles as geomorphological parameters that will affect the ease of building power plant facilities and supporting infrastructures. Also, groundwater is essential as an option for reactor cooling, and West Kalimantan has numerous swamps, causing the surface water to have a low pH [63, 64]. This region has no active volcanoes, and the soil is relatively old, rich in humus, and devoid of mineral content like acrisols, histosols, and fluviosols. The high annual rainfall coupled with numerous wet months, presented by tropical rain forests and wet peat, is very suitable [65]. These rain forests were a Kalimantan landscape icon before the function changed to cultivation, comprising agricultural land, plantations, mining, production forests, and settlements [66]. Consequently, these anthropogenic landscapes can influence the selection of nuclear power plants, land acquisition, and regional spatial planning.

**Figure 4:**
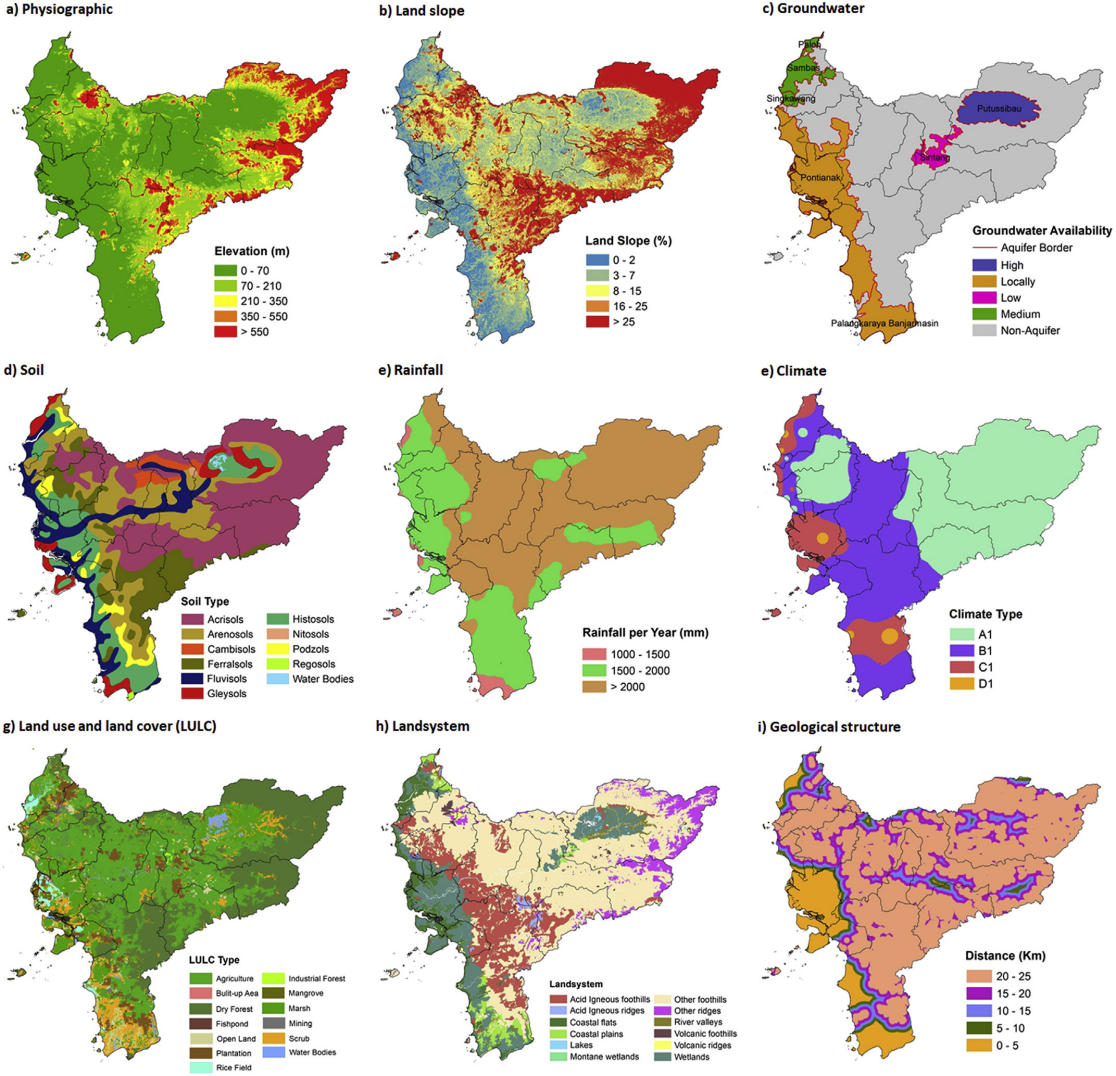
Biogeophysical factors that determinant the suitability of nuclear power plants.

In addition, West Kalimantan has foothills, wetlands, valleys, ridges, as well as coastal plains and flats landform systems. Lands, including foothills, wetlands, valleys, positioned near large rivers such as Kapuas, Landak, Pinoh, Sambas Besar, Sambas Kecil, Ledo, Sebangkau, and Selakau, are fertile alluvial areas for agricultural and plantation activities. Almost all of these rivers lack steep cliffs, and the water flows slowly in a twisting manner, resulting in meandering processes [67]. In coastal areas, the plains and wetlands are downstream of the rivers, and some are affected by tidal interactions, causing the land to be potentially acidic and salty [68]. Also, the geological structure of several locations shows lineaments that divide the basin into several segments and run parallel to each other in a northwest-southeast direction. The lineaments found in Sintang and Sanggau (Lupar Fault) proceed from Kuching to Sekadau, while Adang Fault cuts through Sambas, Sanggau, and Kapuas Hulu. The youngest fault in Singkawang and Bengkayang is of the sinistral type, exists in the Oligocene Sintang Formation, and is aged 23.7–30 million years old. Based on these conditions, coastal areas suitable for nuclear power plants include Sambas Pontianak, Kubu Raya, Kayong Utara, and Ketapang [69]. According to Figure 4, the biogeophysical aspect aims to prevent natural factors from hindering the development and operation of nuclear power plants. Along with socio-economic and disaster considerations, these factors will determine comprehensive and suitable locations.

**Figure 5:**
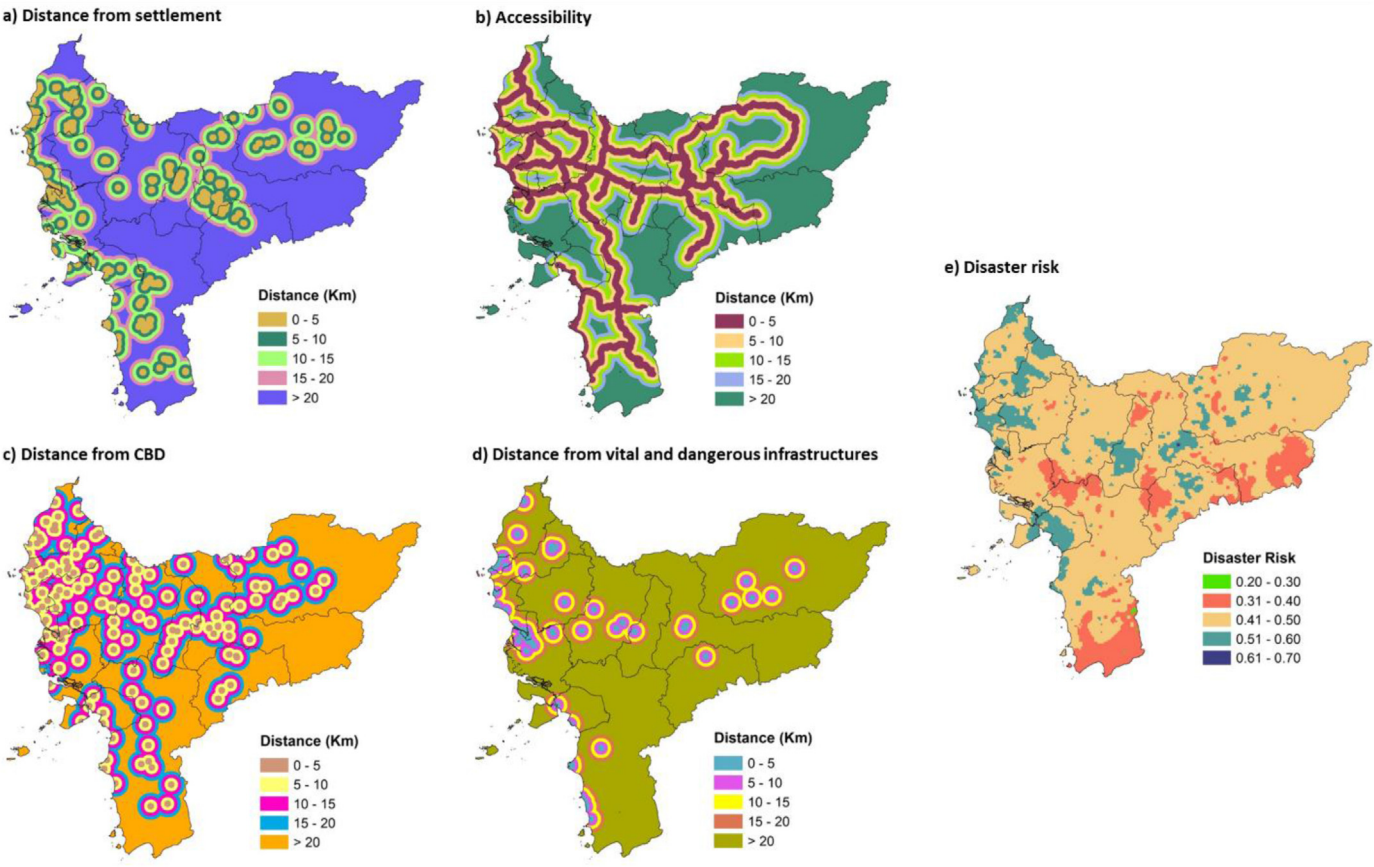
Socio-economic and disaster factors that determine suitability of nuclear power plants.

Humans are the main key to realizing sustainable power plant development projects and operations, hence social impacts require careful attention to avoid disrupting their lives [70]. Nuclear power plants encounter two major challenges, which are public acceptance of atomic energy sources and ensuring safety even in the worst conditions when an incident occurs. Therefore, the settlement location and distance are very important, and nuclear power plant sites should be situated remotely, as although the nuclear power plants are safe and environmentally friendly, the welfare and happiness of society are necessary [71, 72]. In West Kalimantan, many areas are relatively far from the settlements. In addition, reviewing the accessibility is necessary to facilitate the transportation of labor and building materials. The main arterial and collector roads are also crucial parameters, as they can accommodate high transportation loads during the construction of nuclear power plant facilities. Hence, roads are a contributing factor that requires a detailed review, as rural settlements in West Kalimantan are often associated with them, despite the concentration of coastal areas and valley communities [73]. In certain locations, such as the local-administrative capital, there is a Central Business District (CBD), which is the center of government and local economy. Many public facilities, such as schools, jami mosques, government offices, parks, and others, are found around these CBDs [74]. Determining the location of nuclear powers plant considers CBDs, including when district-level regions are newly formed. Meanwhile, the CBD connectivity in this study area is quite close and forms a spatial concentration. Another socio-economic factor to consider is vital and dangerous infrastructures, which, together with disaster factors, play roles in risk management, as shown in Figure 5.

Vital and dangerous West Kalimantan infrastructures consist of existing coal-fired and diesel power plants, oil and gas stations, oil refineries, airports, and harbors, which support the socio-economic aspects of people's lives. In addition to suitability, these infrastructures consider the population size, their distance, and the strength of interregional interactions [75, 76]. Although there are similarities, the distribution is slightly different from CBD and residential locations, which are near the main roads. Knowing their locations ensures disastrous incidents do not have complex impacts and disrupt the operations of the nuclear power plant. Also, identifying potential disasters is necessary to determine the location, as West Kalimantan, for instance, experiences many hazards —land and forest fires, floods, and landslides. During past fire disasters, there were reports of drastic reductions in air quality, and long-range transport of pollutants to Malaysia and Singapore has also occurred [77]. Therefore, disaster and hydrometeorological factors must be considered in determining locations due to threats of global warming and climate change [78].

**Figure 6:**
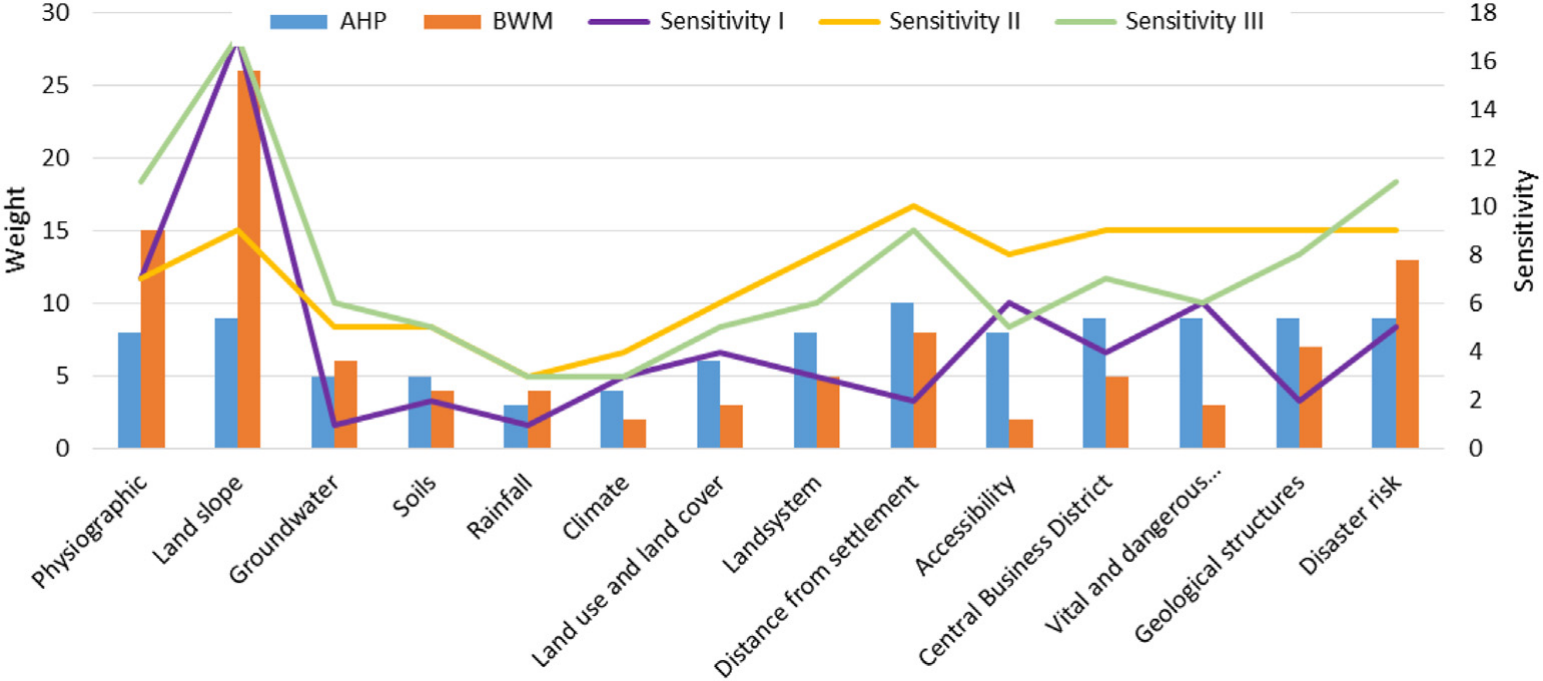
The weight of each parameter from AHP and BWM. Sensitivity I is subtracting from the two weights, sensitivity II is obtained from division, and sensitivity III is the average. The three sensitivity values are displayed as absolute numbers on a scale of 0–100.

**Figure 7:**
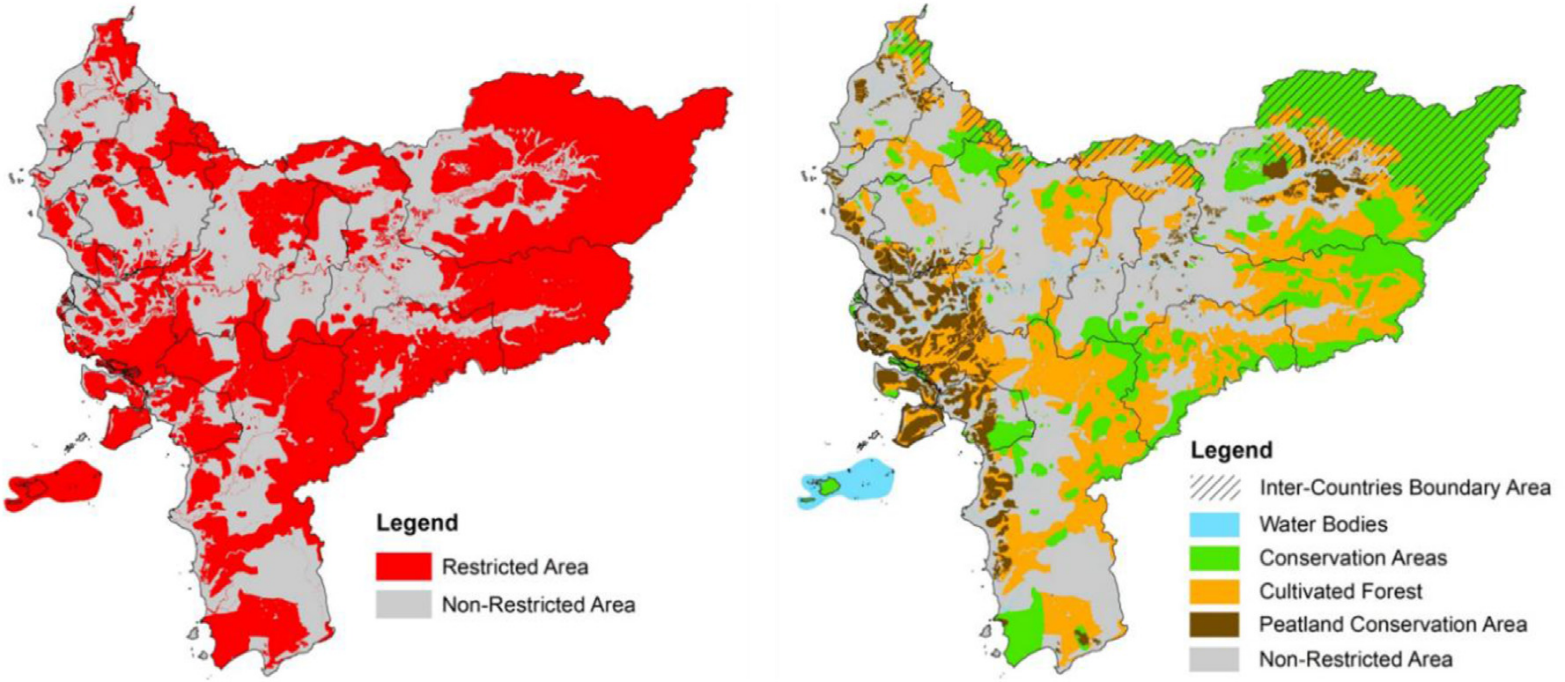
Restricted area of West Kalimantan.

**Figure 8:**
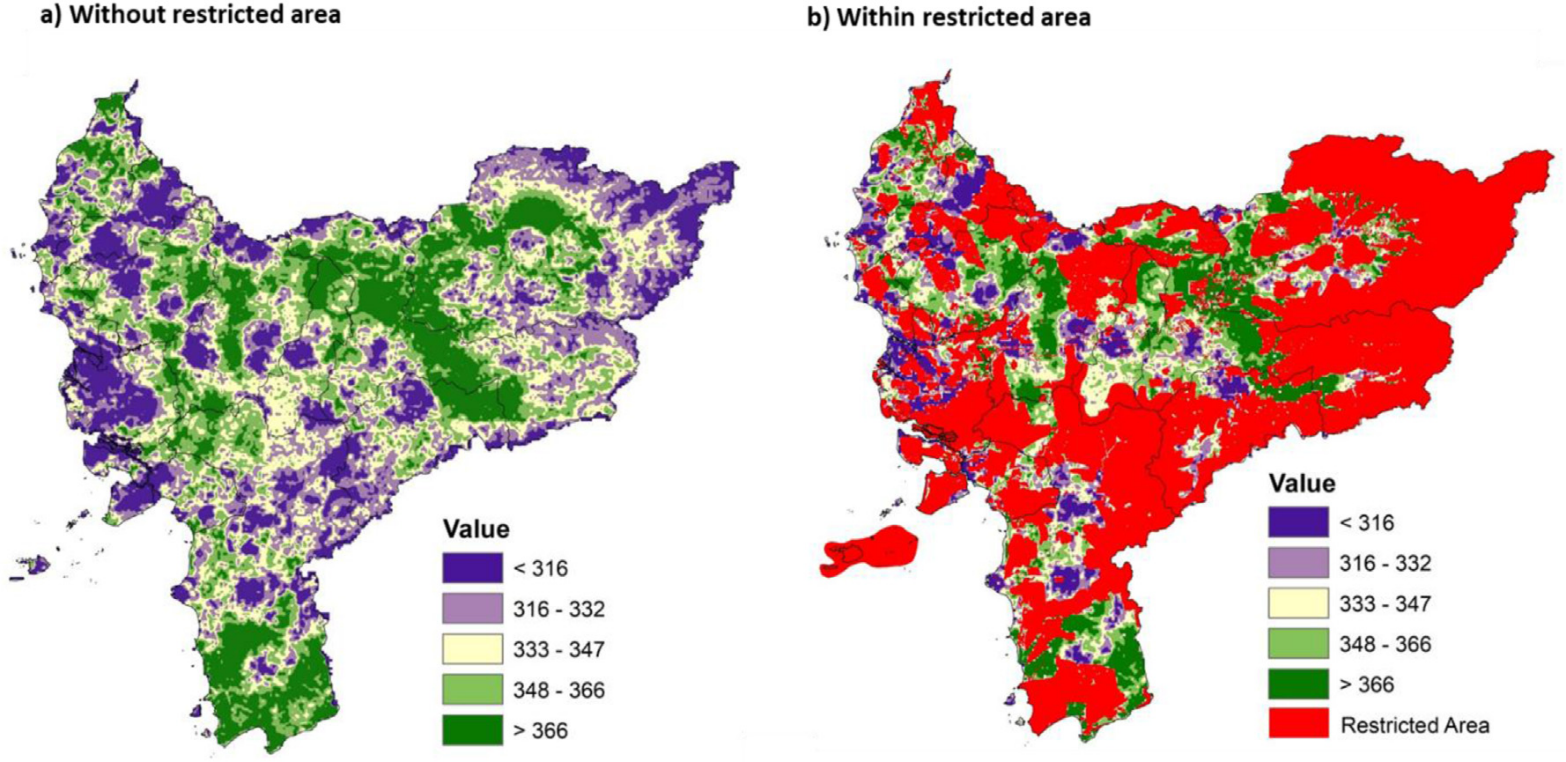
Overlay result that shows the suitable locations for nuclear power plants.

The site for the nuclear power plant can be determined spatially, using the 14 parameters derived from the three factors combined through overlay analysis. These analysis values require reclassification into five suitability classes, namely very high (more than 366), high (316–322), moderate (333–347), low (348–366), and very low (less than 316). This suitability analysis has low level of sensitivity after comparing the AHP results with BWM. The results of sensitivity are presented in Figure 6, both AHP and BWM have the consistency ratio value (CR and Ksi) lower than 0.10, respectively. NPP suitability parameters, especially those from raster data, such as 1) physiographic and 2) land slope have higher sensitivity than others. AHP and BWM have differences in the most priority parameter (best) for the suitability of NPP, these methods also do not consider the worst rainfall parameter. AHP gives weight to biogeophysical, socio-economic, and disaster risk aspects which are not too different. However, BWM prioritizes physiographic and land slope. Both are important indicators for the site because NPP requires a stable area of morphodynamics [79], in addition to population, vital infrastructure, and potential hazards [80]. In this study, several socio-economic parameters are also considered for the suitability of the NPP. Parameter is a guarantee for NPP management and the sustainability of the surrounding community [81]. Establishing the suitability of nuclear power plants must exclude areas determined by the government for the West Kalimantan province. These include prohibited areas, especially those with national and regional regulations, as shown in Figure 7. The restricted area spans 83,72 km^2^ (56.97 %), meaning other non-restricted regions occupy 63249.08 km^2^ (43.032 %). Generally, restricted areas do not always form a matrix but are distributed as landscape fragments, which are very vulnerable to land conversion by the community or local government through spatial regulation changes [82]. As indicated in Table 8. 16,321.66 and 14,321.56 km^2^ were among these exceptions and discovered for the very high and high suitability categories, respectively, for siting nuclear power plants in West Kalimantan. According to Figure 8, the spatial distribution in the interior was greater than near the coast. This is a potential condition, supposing the Indonesian government wants a nuclear power plant that uses a gas reactor cooling system, which is relatively costlier than water [83].

**Table 8:** Suitability for nuclear power plants.

Value	Suitability	Area (km^2^)	%
<316	Very low	10,451.90	16.52
316–332	Low	10,256.25	16.22
333–347	Medium	11,897.70	18.81
348–366	High	14,321.56	22.64
>366	Very high	16,321.66	25.81
Non-restricted area (sub-total)		63,249.08	43.03
Restricted area (sub-total)		83,723.00	56.97
Total		146,972.08	100

Furthermore, the overlay results, excluding the restricted areas, show that although an option to place nuclear power plants in the small islands of West Kalimantan emerged. Meanwhile, an important consideration from this discourse is the unwillingness of the community and local government to live together or coexist with these power plants [84]. Nuclear reactors built for research purposes are located in residential areas in Indonesia and are currently still operating in Java, precisely Bandung, Tangerang, and Yogyakarta. However, the negative stigma about atomic energy still poses a significant challenge [85]. These small islands, some of which are uninhabited, have low and very low suitability classes, and forcing plants to be built in these areas will increase development and operational costs, including the electricity distribution process and worker expenses [86]. Therefore, locating the development and operations on the Kalimantan mainland is more advisable. These processes are increasingly important to support the various activities of West Kalimantan residents, who currently lack electrical energy and are driven to import from Malaysia [87].

## 4 Discussions

**Figure 9:**
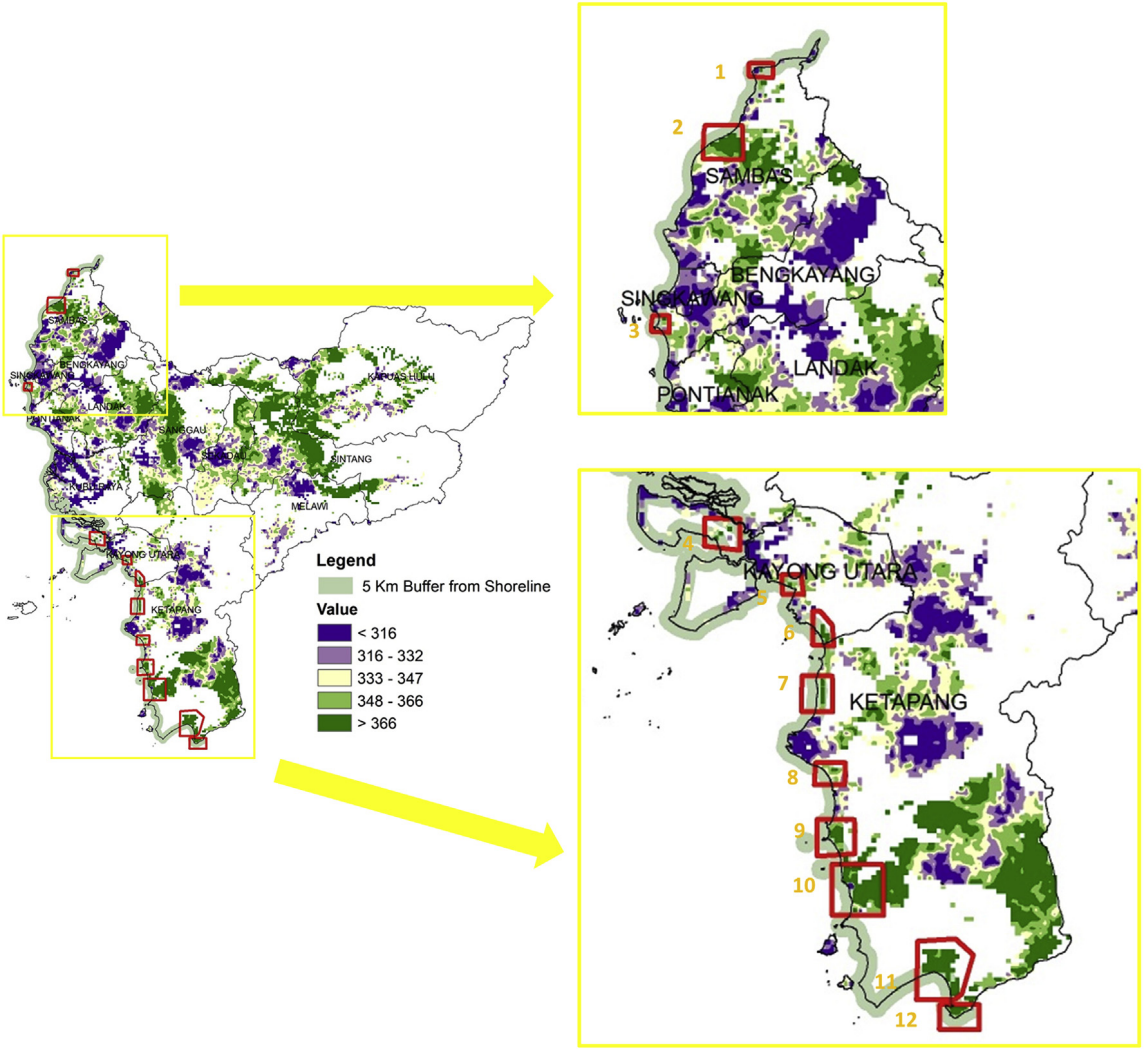
Nuclear power plant priority locations.

The construction of nuclear power plants as a renewable energy source in West Kalimantan prioritizes the use of water for reactor cooling because it is cheaper than gas, is an abundant resource, and can be recycled. Although water is cheaper, it must comply with the quality standards, which occasionally require treatment, such as desalination [88, 89]. Therefore, the location of nuclear power plants prioritizes coastal areas, which must be precisely five (5) km from the shoreline according to site criteria from IAEA [90]. As shown in Figure 9, at least twelve (12) priority locations for a nuclear power plant were discovered in West Kalimantan, precisely two (2) sites in Sambas, one (1) in Bengkayang, one (1) in Kubu Raya, two (2) in Kayong Utara, and six (6) in Ketapang. These coastal areas have the advantage of being located close to main cities such as Pontianak and Singkawang. Also, they support industrial areas that will to operate in the future, despite the agglomeration of residents [91]. Another advantage is the existence of electricity infrastructures from conventional power plants (steam and diesel). Therefore, a nuclear power plant in this area will be the first in Kalimantan and Indonesia and will be able to supply surplus electricity to other provinces and neighboring countries, such as Malaysia and Brunei Darussalam.

Some locations among the twelve (12) priority sites require regional planning. West Kalimantan has two spatial plans at national and provincial levels, 1) Regional Spatial Plan (RTRW 2014–2035) and 2) Zoning Plan for Coastal and Small Islands (RZWP3K 2018–2038) [92,93]. Also, a review of the existing state of locations based on high-resolution satellite imagery data is necessary to avoid social disputes and conflicts of interest, considering many new built-up areas appeared outside the existing spatial plan. This phenomenon is common in Indonesia because it is still facilitated by the legal rules in Law Number 26 of 2007 about Spatial Planning, especially for developing areas [42, 94]. According to the overlap results in Table 9, two priority sites are ideal for the construction and operation of nuclear power plants(sites 9 and 11), both located in Ketapang. These locations are situated far from settlements and built-up areas, allowing some residents to seek relocation carefully.

**Table 9:** Priority location of nuclear power plants with RTRW, RZWP3K, and existing state.

Site		RTRW	RZWP3K	Existing state	Information
1	Sambas	Protected forest; Conversion production forest	Marine Protected Areas, Coastal and Small Islands (KKP3K)	Plantations; agricultural wetlands; settlement	Linear settlement
2	Sambas	Settlement; agricultural wetlands	Catch fishing area	Plantations; agricultural wetlands; settlement	Linear settlement
3	Bengkayang	Settlement	Marine Protected Areas, Coastal and Small Islands (KKP3K)	Plantations; agricultural wetlands; settlement	Linear settlement
4	Kubu Raya	Agriculture; Limited production forest	Marine Protected Areas, Coastal and Small Islands (KKP3K)	Plantations; agricultural wetlands	-
5	Kayong Utara	Settlement	Conservation areas	Plantations; settlement	Linear settlement
6	Kayong Utara	Settlement	Catch fishing area; tourism	Plantations; settlement	Linear settlement
7	Ketapang	Other usage areas	Catch fishing area	Plantations; agricultural wetlands; settlement	Linear settlement
8	Ketapang	Other usage areas	Catch fishing area; tourism; harbor	Plantations; agricultural wetlands; settlement	Linear settlement
9	Ketapang	Other usage areas	Catch fishing area; tourism; harbor	Plantations	-
10	Ketapang	Other usage areas	Catch fishing area	Plantations; agricultural wetlands; settlement	Nucleated settlement
11	Ketapang	Other usage areas	Marine Protected Areas, Coastal and Small Islands (KKP3K)	Plantations; agricultural wetlands; settlement	Nucleated settlement (sligthly)
12	Ketapang	Other usage areas	Catch fishing area; harbor	Pond; settlement	Nucleated settlement

Site-9 is located in Matan Hilir Selatan, far from the population and outside the land-ocean conservation zone. It is flexible for various reasons, such as including other use areas (RTRW) and its application as a fishery, fishing port, and marine tourism (RZWP3K). This site also has the advantage of causing minimal social conflict as it produces minimal alterations in spatial planning. Meanwhile, Site-11 is situated in Kendawangan, allowing for small-scale population relocation, which will affect the residents' livelihoods and result in significant socio-economic impacts [95]. Although there are centrally patterned settlements, only a few exist, where the majority of the population is likely to live as farmers and fishermen. Site-11 includes other use areas (RTRW), marine, coastal, and small island conservation areas (KKP3K), fisheries, and fishing ports (RZWP3K). Another advantage is the site's proximity to Central Kalimantan, especially Palangkaraya, allowing the construction of a plant that can easily distribute electricity to other provinces and the prospective new Indonesian capital city, Penajam Paser Utara, in East Kalimantan [96, 97]. As a limitation, this research requires data detailing and integrating spatial details if we need to mapping a specific area (vicinity site), especially if the assessment will be carried out at the district/city level.

## 5 Conclusions

Spatial-weighted multicriteria analysis shows that West Kalimantan has a very high suitability area for nuclear power plants, which spans 16,321.66 Km^2^ (25.81 %) after deducting the restricted area for development. The majority is located in the coastal areas and valleys of major rivers. After considering the type of reactor enclosure, this study proposed that the development prioritizes coastal areas situated five (5) kilometers from the shoreline. The reasons are that they have abundant potential sources of raw water for cooling materials, are close to development centers and industrial areas, and possess more established electricity networks. Subsequently, the results of the buffer on the shoreline obtained twelve (12) priority nuclear power plants in five (5) districts/cities, namely Sambas, Bengkayang, Kubu Raya, Kayong Utara, and Ketapang. Six (6) of these are located on the Ketapang coast, facing the Karimata Strait and the Java Sea. Meanwhile, the construction and operation of nuclear power plants must consider policy aspects and the existing state to avoid having significant impacts on the surroundings. Consequently, two priority locations were identified as feasible for this project because they did not conflict with the spatial plan (RTRW), coastal and small island management zoning (RZWP3K), and the existing state in West Kalimantan. Although these sites would require relocating the residents, the government only needs to perform these activities on a small scale.

This research is classified as large-scale because the study area entailed one province. The detailing for two priority locations is important to appreciate the environmental setting, including the socio-economic characteristics. Therefore, the construction of a nuclear power plant soon within less than five years, necessitates the government to include these two locations in the revised new spatial plan, especially at the district/city level. The government also needs to inform the public through various media to guarantee their awareness. In the future, site analysis will reveal the detailed suitability of these candidates for the first nuclear power plant in Indonesia, ensuring their presence upholds the values of sustainability.

## Declarations

### Author contribution statement

Heni Susiati: Conceived and designed the experiments; Contributed reagents, materials, analysis tools or data; Wrote the paper.

Moh. Dede & Millary Agung Widiawaty: Conceived and designed the experiments; Performed the experiments; Analyzed and interpreted the data; Wrote the paper.

Arif Ismail: Conceived and designed the experiments; Analyzed and interpreted the data.

Pande Made Udiyani: Analyzed and interpreted the data; Contributed reagents, materials, analysis tools or data.

### Funding statement

This work was supported by Indonesian Ministry of Research, Technology and Higher Education 10.13039/501100010447‘Kemenristekdikti RI’ through RISTEK/BRIN-LPDP Project (14/E1/III/PRN/2020) and PKSEN-BATAN (2020).

### Data availability statement

Data will be made available on request.

### Declaration of interests statement

The authors declare no conflict of interest.

### Additional information

No additional information is available for this paper.
